# Causes, Coping, and Culture: A Comparative Survey Study on Representation of Back Pain in Three Swiss Language Regions

**DOI:** 10.1371/journal.pone.0078029

**Published:** 2013-11-01

**Authors:** Peter J. Schulz, Uwe Hartung, Silvia Riva

**Affiliations:** Institute of Communication and Health, Università della Svizzera Italiana, Lugano, Switzerland; Queensland University of Technology, Australia

## Abstract

**Introduction:**

This study intends to contribute to a research tradition that asks how causal attributions of illnesses affect coping behavior. Causal attributions are understood as the most important element of illness representations and coping as a means to preserve quality of life. The issue is applied to a condition so far often neglected in research on illness representations–back pain–and a third concept is added to the picture: culture.

**Aim:**

The aim of this study is (a) to explore the causal factors to which persons with back pain attribute the further course of their illness, (b) to find out whether the attributed causes are predictors of coping maxims, and (c) to find out whether cultural factors affect attributions and coping and moderate the relationship between the two.

**Methods:**

A total of 1259 gainfully employed or self-employed persons with recent episodes of back pain were recruited in the three language regions of Switzerland. They were asked to complete a structured online interview, measuring among many other variables attributed causes, coping maxims, and affiliation to one of the Swiss micro-cultures (German-, French- or Italian-speaking).

**Results:**

Attributed causes of the illness that can be influenced by a patient go along with more active coping styles. Cultural affiliation impacts on coping maxims independently, but culture moderates the relationship of attributed causes and coping maxims only in two of twenty possible cases.

**Implications:**

The results show that cultural differences can be analytically incorporated in the models of illness representations. Results may help to improve healthcare providers’ communication with patients and plan public health campaigns. The approach to micro-cultural differences and the substantive relationships between alterability of causes and activity in coping may help the further development of models of illness representations.

## Introduction

Effective coping skills allow patients to retain as much of their lives in spite of a medical condition that is chronic or difficult to diagnose or treat. Coping helps to preserve quality of life and may contribute to controlling pain levels. As coping behavior depends foremost on the patients, their lay theories of their illness can be considered a causal agent in their choice of behavior. Lay theories are most often referred to as illness representations. Arguably, their most important dimension is the attribution of the illness to causes. Given the importance of both concepts, a research tradition has developed that asks how causal attributions affect coping behavior [1–2–3–4]. This study intends to contribute to this tradition by applying it to a condition so far almost absent in it–back pain–and by adding a third concept into the picture: culture.

Coping has been a major focus of research in psychology and health sciences for several decades. Folkman and Lazarus [5]–[6] defined it as “the constantly changing cognitive and behavioural efforts to manage the specific external or internal demands that are appraised as taxing or exceeding the resources of the person,” a definition widely accepted in the psychological and medical literature [7]. Coping may be influenced by a number of factors, particularly individual abilities and resources, personality traits, a person’s living situation, the reactions of others, and the advice and education a patient gets from health care providers. It may also be influenced by the way of how someone conceives of an illness, and by preferences and customs prevalent in the society the patient lives in [6]–[7]. The latter two, illness representations and culture, are the focus of this article.

There are many ways of classifying coping behaviors and strategies [8]. A content analysis of research articles finds seven types of coping: avoidance/denial (i.e. ignoring the condition), cognitive reappraisal, visits to one’s doctor, expressing emotion, general problem-focused coping, specific problem-focused coping and seeking social support.

Causal attributions represent the primordial and arguably most influential component in illness representations as they identify the origin of suffering and affect timeline and controllability. Research shows that the perceived alterability of causes mediates their correlation with other dimensions of illness representations. For instance, persons who attributed their asthma to factors beyond their control were found to be less likely to adhere to medication recommendations [9]. Studies on back pain also highlight a beneficial effect of attribution to alterable causes on outcomes [10]–[11], and attribution theory has corroborated the import of perceived controllability beyond health subjects [12]. Therefore this analysis will pay attention to alterability of the causes, too.

According to a review, causal attributions have been classified in so many different ways that comparisons between studies are virtually impossible [1]–[8]. Moreover, a typology of causes into biological, emotional, environmental, and psychological, cited in the same review, offers very broad categories in need of further division, depending on the research questions to be answered.

Findings on the link between causes and coping are as diverse as the measures of causes, and far from consistent. A review of 27 studies found that attributions were related to specific coping strategies; specifically attributions to uncontrollable causes go along with avoidance coping, and attributions to alterable and controllable causes with approach coping and emotion-focused coping [1]. Approach coping implies activity and attention; its opposite is avoidance coping, which stands for passivity and withdrawal [13]. With regard to chronic fatigue syndrome, a recent study found for instance that attributed causes correlated only weakly with coping [14]. In contrast, another study found that causal beliefs predicted help-seeking in a study of older patients with subjective memory complaints [15].

As an addition, we include cultural affiliation in the analysis. Illness representations are understood as influenced by individuals’ perceptions, experiences, attitudes and beliefs, which in turn are affected by social and cultural factors such as family, friends, doctors, access to health care, mass media, and others. In this way, cultural norms and values can affect the way people respond to health-related problems [11–16–17–18–19]. Despite this acknowledgment, the role of culture in illness representations and their consequences has been neglected empirically.

Empirical evidence of cultural influences can be gathered from studies that are to be read against the foil of the western culture of North America and Europe as they look at minorities or other parts of the world [20]–[21]. Better evidence comes from studies comparing different cultures [11–22–23–24]. Most research in this vein takes nation or country as proxy for culture. In some countries, however, distinct micro-cultures exist, the comparison of which can also highlight the impact of culture. One such case is Switzerland, where the three language regions constitute micro-cultures within the larger frame of the Swiss culture. Some research shows that the three micro-cultures do differ with regard to health beliefs and behavior [25]–[26].

Thematically, this analysis is limited to back pain. Back pain represents a significant public health problem; it is the second most common pain after headache, with lifetime prevalence ranging from 60% to 80% [27]. Every second American reported an episode the year before, every fourth in the last three months [28]. Back pain may originate from an injury, disease or various forms of physical stress and may be felt as bone pain, nerve pain or muscle pain [29]. Several treatments are recommended, including various forms of physical therapy, medication, and of alternative and complementary medicine [29]. Despite the many therapies available, no single treatment has been demonstrated to result in patently better outcomes than others and to reduce pain completely [30]. Therefore, we focus on coping rather than health outcomes as the dependent variable. Studies have shown that interventions can change illness representations and positively affect back pain-related outcomes [11], while perceived inability to control the pain can lead to poorer outcomes [10]–[31]. This suggests that causal beliefs on back pain can be the target for interventions, and provides another reason for limiting our study to this dimension of illness representations.

Conceptually, this study is situated within the wider frame of Leventhal’s Common-Sense Model of Illness Representation (CSM), which offers a structure for the analysis of how patients process illness information and how their conceptions of an illness affect health outcomes [16]–[17]. Illness representations have, in the original conceptualization, five components: *identity* (labels for the condition and symptoms), beliefs about *causes* of the condition, its *consequences*, the perceived duration and course of the illness (called *timeline*), and finally, *controllability* (patients’ beliefs about the possibility of a cure or control over their illness) [8].

The CSM holds that these components are all linked with each other, forming a meaningful whole, and that they affect coping responses and health outcomes [32]–[33]. Many studies have found evidence for these links for a variety of different conditions [8]–[32]. That there should be a link between causes and coping is also posited by the CSM [10]–[34]. The CSM makes further claims on an effect of representation on health outcomes. Due to difficulties in diagnosing and treating back pain (see above), these claims are not pursued in our study. Cultural affiliation has hardly ever been included as a variable in studies on the CSM so far, although Leventhal and others have claimed an influence of culture [18].

The article thus pursues two aims. It aspires (1) to study how patients’ beliefs about causes of back pain affect their maxims for coping with the condition in a Swiss context and (2) to detail the role that micro-cultural affiliation with one of the Swiss language regions plays for causal attributions, coping maxims, and the correlation between the two. Aim (1) tests, as a by-product, the applicability of one element in Leventhal’s CSM to a specific condition and a new setting, and Aim (2) its ability to integrate additional independent or intervening variables.

## Methods

### Sample

The data base is provided by a study commissioned by SUVA, a major public insurance company in Switzerland, and the country’s leading insurer against accidents at work and occupational disease. SUVA was interested in the relationship between psychosocial conditions at the workplace and employees’ experiencing chronic back pain, or episodes of back pain, and in particular in how micro-cultural affiliation may affect this relationship. The interest of SUVA was motivated by considering culturally specific interventions to improve prevention and coping.

Mainly for reasons of cost, an Internet survey was projected, drawing on a large telephone-recruited online panel kept by Link Institute, a private survey research company. Link claims that the different economic branches are adequately represented in the panel, implying that a sample of (self-)employed persons drawn from the panel can be representative of the Swiss workforce. Sampling and fieldwork were done by the Institute.

The Link Institute invited a total of 7793 member of their online panel to participate in the survey. Invitations were first sent out January 19, 2011 and repeated the next day and a third time a week later. Polls were closed February 4, 2011. Of the 7793 invited persons, 2785 ( = 35.7%) declared their willingness to take part in the survey. They were contacted about the survey.

Inclusion criteria were (a) having had an episode of back pain or worse within the past year, (b) being gainfully employed or self-employed at the time of the survey, and (c) willing to cooperate. Of the 2785 persons contacted, 1300 (47%) had not had back pain. Among those with back pain, 190 (13%) were not (self-)employed. Among the 1295 workforce contacts, 36 declined cooperation, leaving 1259 persons. They were asked a detailed online questionnaire.

The survey was conducted in one of the three official languages of Switzerland (French, Italian, German), depending on the respondent’s preference. The questionnaire was drafted by staff members at the Institute of Communication and Health in Lugano in German and then subjected to forward-backward translation. That means the German version was translated into French and Italian, and then back into German by different translators. Original and backward translation were compared and necessary adjustments made. The online versions were pretested, especially for user-friendliness and comprehensibility.

### Measures

The survey contained a large number of questions on matters such as

- perceptions of workplace conditions (especially stress),
- patient representations of back pain,
- coping directed at the pain,
- coping directed at stress,
- severity of back pain,
- restrictions inflicted by the pain condition,
- therapies tried and their success,
- medical consultations,
- and demographics.

From among these groups of variables, this article uses only causal attributions (as one dimension of patient representations of back pain), coping directed at the pain and the demographic variable of micro-cultural affiliation.

Some studies attempt to develop anad validate general measures for these concepts, but others use condition-specific measures (for a brief discussion of this see [1]–[35]). As we were interested in the specifics of representations of back pain, we opted for constructing our own measures. The scales derived from factor analysis of these measures showed acceptable reliability (see below).

Two different types of causes can be distinguished, the original cause that first made patients’ back hurt and, secondly, influence factors on the further course of the illness. This echoes the distinction between causal (responsibility for problem) and recovery beliefs (responsibility for solution) [36], only that we include in the latter also the possibility of a worsening problem. As presumably the recovery/worsening beliefs are more closely linked with coping, we deal with these rather than the attribution of original causes. Influence factors were measured in two questions, “What does it depend on whether your back pain, in the long term, will get better or at least not worse?” and “Besides the long-term influences, there may be short-term circumstances that trigger the onset or a special worsening of your back pain. How strongly do the following circumstances influence your back pain?” Answers were given on 0 = “Not at all” to 7 = “Very strong influence” scales. Eleven items were offered in the first question, nine in the second. They were taken together in all subsequent analyses.

Coping measures usually either inquire about behavior or about strategy [4]. Both are somewhat problematic in our context. Behaviors will be restricted or encouraged by circumstances and therefore be a result of people’s ideals and preferences on one hand, and all kinds of restraints. And “strategy” might be too demanding a concept for what is actually anchored in people’s minds. Therefore we tried to capture coping maxims, that is: simple rules for behavior and intentions vis-à-vis a back pain condition. Twelve statements were offered, preceded by “To what extent do the following statements apply to you?” Answers were given on 0 = “Not at all” to 7 = “Applies completely” scales.

Micro-cultural affiliation with one of the three Swiss lingual groups was determined by the language chosen for the interview.

### Data Analysis

All twenty items of perceived causal agents were subjected to a factor analysis, and so were the twelve statements of coping maxims. To identify micro-cultural differences in cause attribution and coping maxims, average factor scores were computed, ANOVAs were run and pairwise significant differences ascertained by Scheffe post hoc tests. For assessing the link between perceived causal factors and preferred coping maxims as well as the possible moderator role of culture, multiple stepwise regressions were run with agreement to one of the coping maxims as dependent variable and ascribed cause, dummies for micro-cultural affiliation, and interactions between the two as independent variables.

### Ethics Statement

The research has been approved by the authors’ institutional review board of Canton Ticino (Bellinzona, Switzerland). The IRB has approved this study on 06/07/2010 with the following project number: “IHC-SUVA”. Written informed consent was obtained for each participants included in this study.

## Results

Somewhat more respondents were male than female; most were married and between 30 and 49 years old. In an intentional deviation from the real distribution, 56% were German-speaking, 26% French-speaking and 18% Italian-speaking. This comes down to a small over-representation of French-speaking and a large over-representation of Italian-speaking persons. It was done to strengthen the basis for analyses especially of the Italian-speaking micro-culture. As no marginal results for the total Swiss workforce play an important role in this article, no weighting of cases was applied.

The low back was the most frequent location of pain, followed by the neck, and still more than a third of respondents complained of pain in the shoulder. Table 1 summarizes some demographic and illness characteristics of the sample.

**Table 1:** Demographic and illness characteristics of the sample.

Demographic variables	N	%	Pain-related variables	N	%
Gender			Localization of backpain**		
Male	699	56	Low back	927	74
Female	560	44	Neck	564	45
Marital status			Shoulder	473	38
Married	711	56	Other	48	4
Other/unknown	548	44	Intensity of back painat interview		
Age			Severe (6 or 7 on0–7 scale)	28	2
29 or younger	205	16	Medium (2–5)	664	53
30–49	740	59	Light (0–1)	569	45
50 or older	314	25	Chronicity* (N = 1249)		
Educational level(n = 1238)			Yes	684	55
Elementary	62	5	No	565	45
Vocational	515	42			
Higher education	661	53			
Swiss region					
German	703	56			
French	328	26			
Italian	228	18			

* Have had back pain for more than 3 months.

** More than one answer.

N = 1259 unless otherwise noted.

### Factor Analysis of Attributed Influence Factors (Independent Variables)

Factor analysis identified five factors in the list of 20 causes, which together explained 67% of the total variance. Cronbach’s α coefficients indicate satisfactory consistency (ranging from 0.69–0.79) of the resulting item sets for each factor. Two items, “Ample sleep” and “My personal susceptibility,” both from the question on long-time influence factors, could not be subsumed to any of the factors and are excluded from further analysis. Table 2 shows the items, the labels given to the factors and the factor loadings retained for analysis if >0.4 (except for one item included in Factor 5).

**Table 2:** Factor analysis of attributed influence factors (causes) on the course of a back pain condition.

	1	2	3	4	5
	Emotion and mood	Job stress	Weather	Physician’s influence	Considerate physical activity
My mood (short term)	.736				
Unusual psychological strain on the job (short term)	.626				
Unusual pressure at home (short term)	.506				
The support I get from my personal environment (long term)	.487				
The full moon (short term)	.460				
Fate, whether I am lucky or not (long term)	.429				
My fatigue (long term)	.430				
The pressures on the job (long term)		.783			
Unusual physical strain on the job (short term)		.543			
Draught (short term)			.824		
The weather (short term)			.817		
My doctors’ competence (long term)				.819	
The regularity with which I go and see my doctor (long term)				.677	
Compliance with doctor’s orders (long term)					.672
The regularity with which I exercise (long term)					.501
Physical exercise (short term)					.422
Improper movements and/or postures					.351

Seven items load on the first factor. They include psychological or emotional matters on the job and at home, and the items on mood, fate, fatigue and the full moon, which are all related with mood. Therefore the factor is labeled “Emotion and mood”. All the items belonging to this factor were overall rated as weak forces on the course of respondents’ back pain.

The second factor includes long-term pressure on the job and short-term unusual physical strain there. It is termed “Job stress.” The third factor unites draughts and the weather, and we use the latter as label. The fourth factor collects items on the influence of one’s physician on one’s back pain. One item is physician-centered (competence), the other patient-centered (regularity of consultation).

A third physician-related item (adherence to therapy) loads stronger on the fifth factor, which foremost considers physical exercise. The adherence item is well placed there as therapy suggestions are likely to have included instructions on proper physical activity. We label the scale “Considerate physical activity,” and aside from the exercise and adherence items, those on wrong movement or posture and on recognizing one’s limits belong here, thus including not only exercise but also attention to one’s movements beyond exercise.

The ascribed causes, respectively the factors, vary with regard to alterability; some can be willingly influenced, others cannot. “Considerate physical activity” is almost completely under control of the patient, while the weather is completely beyond his or her control. Between these poles, control is relatively high for “Physician’s influence” as a patient has more or less complete control over the regularity of visits with a doctor and can always opt to not further consult a physician considered incompetent. Control is lower for “Job stress” as efforts to change work routines and organization concern others and relate to material interests, and are therefore hard to achieve). Control is still lower for “Emotion and mood,” which most people in most situations experience as something beyond their control.

### Factor Analysis of Coping Maxims (Dependent Variables)

Factor analysis revealed four dimensions in the list of coping maxims, with items with factor loadings >0.4 retained for analysis (Table 3). The four factors together explained 54% of the total variance. Cronbach’s α coefficients indicate satisfactory consistency (ranging from 0.60–0.72) of the resulting item sets for each factor.

**Table 3:** Factor analysis of coping maxims.

	1	2	3	4
	Aspiration to improvement	Acquiescence in the condition	Continuation of one’s former life	Acceptance of blame
I have concrete goals for the future, such as being able again to lift my child or to ride horseback	.748			
It is my aim to be able to do all that I could do before my troubles began	.707			
Even if people find it odd, I still do things for my back (such as sitting on a ball rather than a chair)	.700			
I can only fully take part in life again when I get rid of my back pain		.770		
As no one can see my pain, people assumedly think I simulate it		.667		
When I am in pain, it feels good to be able to talk to people about it		.631		
That my back pain gets better is a priority; my job has to take second place		.541		
I more or less have got used to my back pain; they have become part of my life			.733	
I mostly try not to have people notice I am in pain			.701	
I should avoid movements that can worsen my pain			.465	
I myself am to blame if my back pain does not get better				.824
I feel a bit guilty for not doing more for my back				.801

The first factor can be called “Aspiration to improvement” as it includes the two items on goals to get better as well as the willingness to act as the condition demands–even in the face of being looked upon. The items formulate what the person with back pain aims at or does to cope with the condition. They indicate an active striving for getting better and getting along. Comparisons between the cultural groups show that German- and Italian-speaking respondents agreed with this maxim more than the French-speaking.

The second factor stands for “Acquiescence in the condition” as it includes items on the limitations the condition sets on the respondent (not being able to fully participate in life and the necessity of the job having to stand back to the pain) and on additional problems that come along with the illness (the merits of talking about the condition, which can be considered indicative of a perceived lack of social support and understanding, and the fears of appearing to simulate, thus adding injustice to the pain). Agreement to the items emphasizes the suffering inflicted by the pain and related circumstances. In contrast to the pro-active moves against the pain expressed in the first factor, the second formulates passive reactions. One could almost say that agreement to the maxims in this dimension comes down to the absence of coping. The four items on “Acquiescence in the condition” are those with the lowest agreement (scale average), indicating that majorities among respondents rejected this maxim. Comparisons between the cultural groups show that Italian-speaking respondents agreed with this maxim more than the French-speaking and clearly more than the German-speaking (see Table 4 below).

**Table 4:** Average factor scores of attributed causes and coping maxims by cultural group (bivariate analysis).

	German-speaking(n = 580)	French-speaking(n = 256)	Italian-speaking(n = 160)	F	p
Attributed causes					
Emotion and mood	−.12^a^	.22^b^	.07^ab^	12.885	<.001
Job stress	−.11^a^	.16^b^	.14^b^	10.245	<.001
Physician	−.14^a^	.17^b^	.24^b^	19.591	<.001
Weather	−.08^a^	−.06^a^	.40^b^	18.696	<.001
Considerate physical activity	.01^a^	−.04^a^	.03^a^	0.529	ns
Coping maximes	(n = 583)	(n = 276)	(n = 155)		
Aspiration to improvement	.09^a^	−.19^b^	.10^a^	8.224	<.001
Acceptance of blame	.12^a^	−.29^b^	.00^a^	16.463	<.001
Continuation of former life	−.06^a^	.08^a^	.12^a^	3.085	<.05
Acquiescence in the condition	−.16^a^	.19^b^	.31^b^	21.218	<.001

Df = 2, 993 for the causes; df = 2, 1011 for the coping maxims. Superscripts indicate significance of pairwise comparisons in rows. Same letter means no significant difference. Based on Scheffe post-hoc tests.

The third dimension appears to overlap in substance with the second as one of the items formulates acquiescence (having got used to the pain) and another one resonates the social fear of being stigmatized (trying not to show the pain). The item on avoiding certain movements also loads strongly on this dimension, which appears to have the notion of “Continuation of one’s former life” as a common element. It indicates a position that one is able, with some care, to convince oneself and others that nothing much is amiss. The idea clearly suggest passivity. Comparisons between the cultural groups show that Italian-speaking respondents agreed with this maxim slightly more than the French-speaking, and these clearly more than the German-speaking members of the workforce.

The fourth dimension straightforwardly focuses on responsibility, covering the items that oneself is to blame if the back pain does not get better, and of feeling guilty for not doing more. Both items clearly represent an attribution of the responsibility for coping or improving to the afflicted persons themselves. As such it would fit to the first dimension, Aspiration to improvement, as a coping maxim that indicates activity, or provides a basis for activity. We call it “Acceptance of blame.”

### Cultural Differences in Attributed Causes and Coping Maxims

The three Swiss micro-cultures did not differ in ascribing the further course of their pain to “Considerate physical activity.” The group averages of the factor scales (Table 4) are similar (i.e. close to the total mean of zero) for all three cultures. Swiss Germans uniformly ascribed the least influence on back pain to the three other potential causes (factor scores are all below zero). In contrast all scores for the Italian-speaking and three of four for the French-speaking are above average.

**Figure 1:**
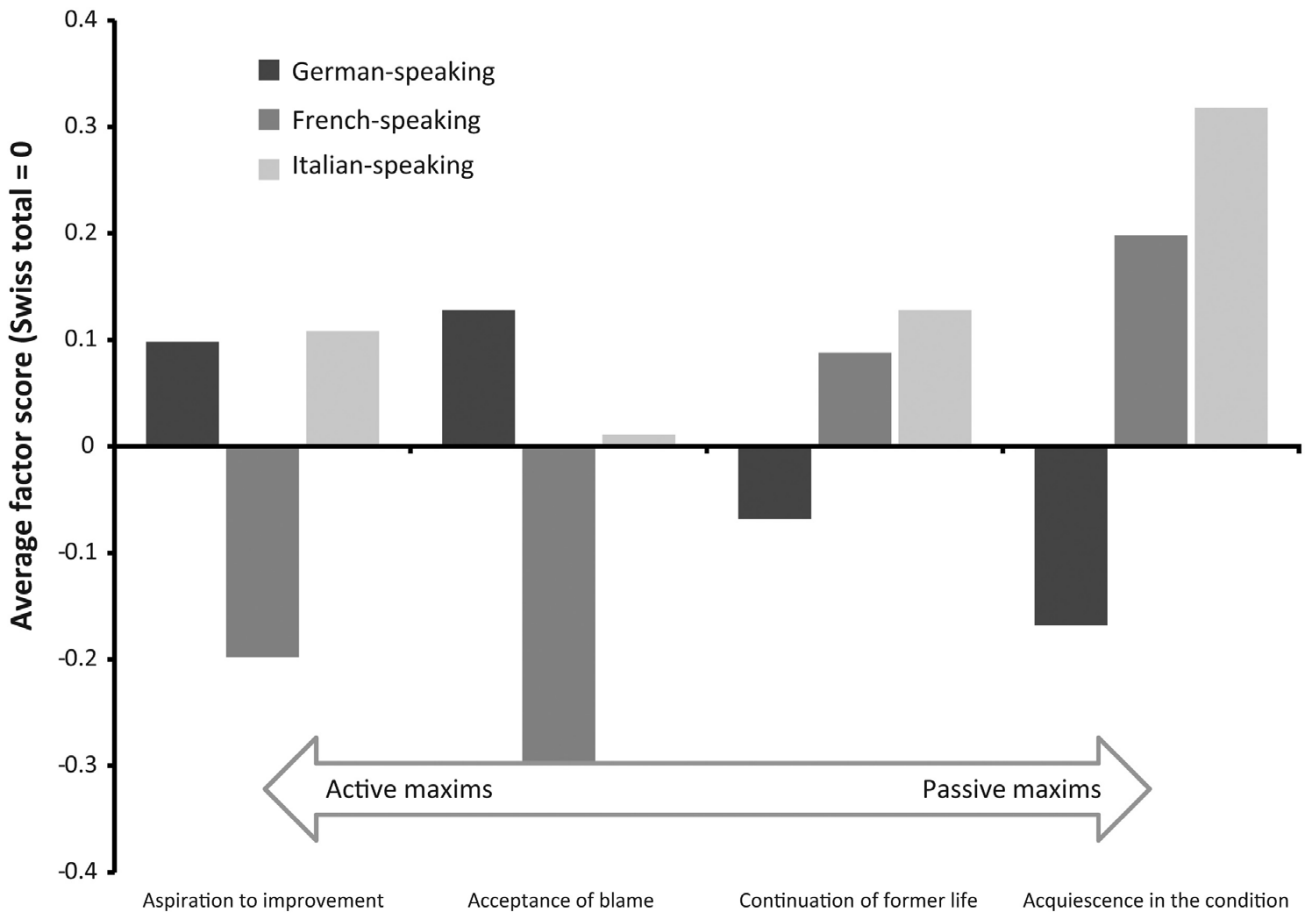
Average factor scores of coping maxims by cultural group (bivariate analysis).

Cultural differences in coping maxims were already mentioned in some detail. Three patterns, however, need to be summarized. The German-speaking Swiss members of the workforce agreed above average to the maxims that indicate active coping, and agreed below average to those indicating passivity in coping. The French-speaking mirrored this with different signs: below-average advocacy of active coping and above-average of passive orientations. This is a clear and complete contrast in the coping maxims between the two groups. The Italian-speaking strangely agreed to the active maxim of aspiration to improvement as decidedly as the German-speaking. With regard to acceptance of blame, their agreement was lower than among the German-speaking but higher than among the French-speaking. But they also agreed to the passive maxims more intensely than the French-speaking. Figure 1 illustrates the different patterns.

### Effect of Attributed Influence Factors on Coping

For assessing the link between perceived causal factors and preferred coping maxims as well as the possible moderator role of culture, regressions were run. In a first analysis, causes, two culture dummies, and all possible interactions were entered into regressions stepwise. This yielded models that showed multiple but by far not uniform effects of ascribed causes on coping maxims, some effects of culture on coping, but only very few significant interaction effects. As the inclusion of interactions did not improve the R^2^ of the models, but weakened some of the links between causes and coping, we included in the final regressions only those interactions that had shown independent effects of both culture and attributed cause, as well as two more for which a significant interaction had emerged while culture and cause alone had been unrelated to coping maxims. The final models are shown in Table 5.

**Table 5:** Effects of attributed causes and culture on coping maxims (regression analysis).

	Dependent variable: Maxims for coping with a back pain condition			
	Aspiration to improvement	Acceptance ofblame	Continuation offormer life	Acquiescence in the condition
Attributed causes				
Emotion and mood	.074	.091 #	−.060	.234***
Job stress	−.076	−.055	.181***	.056
Physician	.143**	−.057	.084*	.313***
Weather	−.012	.038	.132**	.019
Considerate physical activity	.316***	.230***	.085*	−.079*
(Adj. R^2^)	(.153)	(.066)	(.115)	(.278)
Culture				
French-speaking	−.153***	−.163***	.023	.059*
Italian-speaking	−.014	−.032	.001	.093**
(Adj. incr. R^2^)	(.017)	(.023)	(−.001)	(.007)
Interactions				
French-speaking X				
Emotions and mood	.034	.042		
Job stress		−.080		
Physician	.038			
Considerate physical activitiy	−.044	.043		
Italian-speaking X				
Emotions and mood				.001
Job stress	.138***			.076#
Physician				−.021
Weather	−.092*			
Considerate physical activitiy				−.005
(Adj. incr. R^2^)	(.010)	(−.001)		(.001)

(#p<.10, *p<.05, **p<.01, ***p<.001).

If the relationships are looked at from the perspective of the ascribed causes, belief in an effect of *considerate physical activity* was strongly and positively linked to the active coping maxims of aspiration to improvement and acceptance of blame. The belief was weakly related with continuation of one’s former life, and weakly and negatively to passive acquiescence. Belief in the most alterable cause clearly went along with active coping maxims. Respondents who believed in an effect of their *physician* on the condition (seeing him regularly as well as her capabilities) claimed high aspiration for improvement on the active side and also attested, on the passive side, to high acquiescence as well as high inclination to go on as before. Respondents who saw *stress on the job* as causing their back pain advocated coping maxims that come down to the desire to continue their former life, that is: hiding the condition from one’s own inner eye as well as from others. Finally, belief in the unalterable effects on the pain of *emotions and moods* and the *weather* went along with acquiescing in the condition and claims for a continuation of one’s former life respectively, that is to say: with the more passive maxims for coping.

Most of the high and significant regression betas are either indicative of a link between attribution of the further cause of one’s pain to alterable causes and active coping maxims, or between unalterable causes and passive coping. The notable exception is the strong link between belief in physician influence and acquiescence. Table 6 shows the relevant betas (taken from Table 5) in an order that illustrates the link between the alterability of attributed causes and activity/passivity in coping.

**Table 6:** Alterable causes and active coping are correlated, as are unalterable causes and passive coping.

		Dependent variables: Maxims for coping with a back pain condition					
		Passive		←–––––––––––→		Active	
		Acquiescence in the condition	Continuation of former life		Acceptance of blame		Aspiration to improvement
Independent variables: Attributed causes							
Weather	Unalterable		.13**				
Emotion and mood	|	.23***					
Job stress	|		.18***				
Physician	|	.31***	.08*				.14**
Considerate physical activity	|Alterable	−.08*	.09*		.23***		.32***

Table shows significant betas from Table 5.

### Effect of Culture and Interactions of Culture and Causal Attributions

Being French-speaking was related to less advocacy of aspiration to improvement and acceptance of blame. This difference had already emerged in the bivariate analysis; it evidently holds net of the influence of attributed causes on coping. Being Italian-speaking increased acceptance of acquiescence in the condition as a maxim for coping. This also was shown in the bivariate analysis and confirms the relationships shown there.

Where cultural affiliation was associated with coping, interaction effects of culture and perceived causes did not emerge. That is to say, the cultural groups differ in a few aspects in the coping maxims they advocate, but these differences do not moderate the association of ascribed causes and coping maxims.

Moderation was observed in two other cases. The perception that one’s pain was affected by stress on the job was overall not related with aspiration for improvement; the insignificant beta was negative. Affiliation with the Italian-speaking culture in itself did not affect this maxim either, but both together increased the advocacy of an aspiration for improvement. That is to say, while for the rest of the country attribution of back pain to job stress was unrelated to aspiration for improvement, it was positively related to this aspiration of the Italian-speaking. The more they saw their pain affected by stress, the more did they hold that aspiration to improvement was a right way of coping.

The second case of moderation concerns the weather. Attribution of the pain to the weather can be expected to be negatively related with aspiration to improvement as the weather cannot be influenced. They were overall unrelated though, but the insignificant beta was negative indeed. If the regions are regarded separately and significance is taken into account, attribution to the weather and aspiration to improvement were not associated among the German- and the French-speaking. The Italian-speaking, however, are different: the more they ascribed their back pain condition to the weather the less did they favor aspiration to improvement as coping maxim.

With the German-speaking as reference group in Table 5, nothing can be said about the significance of differences between the French- and the Italian-speaking. Running the regressions with the French as reference group shows significant differences with regard to being Italian-speaking as well as the two interaction effects.

## Discussion

All in all, the picture that emerges is a rather coherent one. Swiss back pain patients’ conception of the influence factors on their condition are meaningfully related with the coping maxims they uphold. The more they see an influence of considerate physical activity, the more they aspire to improvement in their coping. The same goes for attributing the further course of the condition to one’s physician, an attribution that in turn also increases acquiescence as a coping maxim. Acquiescence is also increased by the perception that one’s pain is affected by one’s emotions and mood. Attribution to the weather and to the stress on the job enhance continuing one’s former life as a coping maxim. Leaving details and a notable exception aside, attribution of one’s pain to causes that can be affected goes along with more active coping, while attribution to causes not subject to human influence strengthens passive coping maxims. It is a symptom of reasonableness in people’s efforts to make sense of illness. The noteworthy exception to this is the effect of perceived physician influence (relatively easy to affect) on acquiescence (passive).

As to cultural influences, the German-speaking Swiss are less inclined than their compatriots to assign a strong effect to the causes we asked about. As to coping, we find a clear preference of the German-speaking for more active coping and of the French-speaking for more passive coping. With regard to active coping, the Italian-speaking resemble the Swiss Germans, but for passive coping maxims, they outdo the French-speaking. Finally the study demonstrated the existence of interaction effects, that is a moderation of the correlation of causal perceptions with coping by culture. There were only two examples of this (of 20 possible), but they corroborate the necessity of accounting for this possibility in respective studies.

It is clear from these results that the one element of the CSM studied here is applicable to back pain, to a Swiss context, and that it is able to accommodate micro-cultural influences. Some of the findings on culture deserve a closer look.

The below-average scores for the German-speaking Swiss on the scales that measure attribution of the pain to causes might be indicative of a tendency toward more differentiation, or put negatively, towards less open-mindedness with regard to possible influence factors on diseases. Differentiation would mean a strong belief in some possible causes, and rejection of many others, possibly based on criteria of scientific rationality. Open-mindedness would mean to ascribe strong effects across a number of causes without taking the trouble to think about whether my physician or the weather is more consequential for my back pain. That Swiss Germans name fewer causes is corroborated by averaging the original rating across all 20 items. The score for German-speaking is 3.20, with the French-speaking significantly higher at 3.55 and the Italian-speaking at 3.67 (F = 21.251, df = 2, 1255, p<.001). This has, maybe, a weak ring of Germanic severity vs. Latin light-heartedness. For the Italian-speaking attribution of back pain to job stress is associated with the coping maxim of aspiration to improvement. This could indicate a meek position of the German- and French-speaking respondents towards changing conditions at the workplace if they cause pain, a position not shared by the Italian-speaking.

A curious case is the weather, which was ascribed much more influence on back pain by the Italian-speaking than by the other two cultural groups. At the first glance this appears somewhat absurd, given the fact that the Italian-speaking Swiss live in the climatically privileged region of Ticino. It makes more sense if we reconsider we studied perceptions. Maybe the good weather in Ticino makes back pain patients more alert to the consequences of bad weather conditions just because they are rarely occurring.

But it is not only aspects of the micro-cultural differences that deserve attention. Respondents who saw stress on the job as causing their back pain advocated coping maxims that come down to the desire to continue their former life, that is: hiding the condition from one’s own inner eye as well as from others. That might have to do with job requirements, in particular the requirement to hide any restriction to one’s productivity and capacity.

Workplace conditions, especially stress, were one focus of the wider study, of which the reported analyses in this article are a part. If they are taken into account, the picture becomes rather complex. The present analysis shows, for example, that the Italian-speaking Swiss (as the French-speaking) perceive a stronger influence of job stress on the further course of their back pain than the German-speaking. Other analyses (as yet unpublished) from this project show that Swiss-Italians report a lower occurrence of stressors on their job, but evaluate those that happen as more of a burden than the other two micro-cultures. And if independent measures of stress and back pain severity are linked, no correlation emerges for the Italian- in contrast to the German-speaking persons. Put colloquially, the Italian-speaking respondents report fewer stressful events at work, but they suffer more from them. They believe stress causes back pain, but “objectively” the two are not linked. And finally, interpreting the interaction effects, they are prepared to active coping behavior if they think their back hurts due to job stress. We will not make any effort here to untangle this maze, but it suggests that phenomena of perception and objective states both have a part in affecting illness representations. Their respective parts are in in need of further inquiry.

Micro-cultural differences in illness representations need to be traced back to more general qualities of the three language groups. That micro-cultures have a potential to affect individuals is widely recognized [37]–[38]. Sociological and socio-psychological literature finds Nordic and German cultures to be more autonomous and individualistic in the transitory stages in their lives than Latin and Mediterranean cultures [39]. Active coping with regard to chronic health conditions can be interpreted as an expression of German autonomy and a sense of the possibility of helping oneself before one asks others for help. In contrast, passive acquiescence, which included maxims of seeking social support in coping, stand for Latin collectivism and a tendency in Latin cultures to put the blame elsewhere (government institutions in particular) rather than feeling oneself responsible.

The finding on the connection between alterable causes and active coping as well as the hints about micro-cultural differences suggest further investigation in designs that overcome the present study’s limitations. Including other elements of Leventhal’s complex theorization and variables such as age, socioeconomic status, clinical severity, and treatment history are advisable. Among the elements to be considered are the attributed primordial first causes of the pain, perceived consequences, coping behaviors (rather than the maxims studied here), medication adherence, and treatment.

The study did not include variables likely to affect the attribution of causes to back pain and coping maxims, such as gender, age, type of job (blue collar or white collar), or the location of back pain (neck, shoulder, lower back). As this study was intended to make a first step into the ways micro-cultural affiliation can affect illness representations and coping, there was no necessity for modeling that includes all variables that might play a role. Rather than including all these, we chose to focus on the key variables. A somewhat more serious limitation is the lack of measures of other medical conditions, in particular depression and overweight/obesity. Back pain is associated with both [40]–[41], and both might affect coping maxims, depending on for instance on patients’ self-efficacy with regard to changing the conditions.

From a practical and clinical point of view, this study shows that patients’ beliefs about the cause of their back pain symptoms are important determinants of how they manage these symptoms. Coping strategies are as specific as patients’ specific representations of back pain. Health care providers should be aware of this diversity in representations and its impact on willingness to adopt certain coping behaviors, for instance that the French-speaking Swiss are more in need than their compatriots to be convinced that they can do something against back pain.
